# Supplementary material for: Potential Risks of PM2.5-Bound Polycyclic Aromatic Hydrocarbons and Heavy Metals from Inland and Marine Directions for a Marine Background Site in North China
Source: Toxics. 2022 Jan 11;10(1):32. doi: 10.3390/toxics10010032 (PMC8779893; doi:10.3390/toxics10010032)
Supplement: Supplementary file 1 [file toxics-10-00032-s001.zip › toxics-1498258-supplementary.pdf]

toxics

MDPI

Supplementary Materials: Potential Risks of $PM_{2.5}$-Bound Polycyclic Aromatic Hydrocarbons and Heavy Metals from Inland and Marine Directions for a Marine Background Site in North China

Qianqian Xue, Yingze Tian, Xinyi Liu, Xiaojun Wang, Bo Huang, Hongxia Zhu and Yinchang Feng

Text S1. The backward trajectory analysis

To identify the potential directions of air masses and the effect of different transport routes on particle chemical compositions in TI, the backward trajectory was calculated during the sampling period. Five groups of air masses were identified according to their transport directions and area of travel (Figure 3). Cluster 1 (the southeast direction: SE) accounted for 17.7% of the total trajectories, originating from the Huang Sea and passing inland over Yantai, and clearly showed shorter transport patterns. Cluster 2 (the northern direction: N) accounted for 11.8% of the total, beginning in Inner Mongolia and passing through the Liaoning. Cluster 3 (the northwest direction: NW) accounted for 14.0% of the total, originating from Outer Mongolia and crossing over Inner Mongolia and Liaoning province, constituted long-range transport patterns. Cluster 4 (the west-northwest direction: WNW) accounted for 15.0% of the total, beginning in Outer Mongolia and passing through the Inner Mongolia and the Beijing–Tianjin–Hebei region. Cluster 5 (the southwest direction: SW) accounted for 41.6% of the total, originating in the Bohai Sea before arriving at TI.

Text S2. Diagnostic ratios

Hopanes and steranes are emitted by lubricating oils in gasoline and diesel vehicles, while hopanes are also present in the smoke of coal combustion [1–4]. Configurations of hopanes can be employed to identify fossil fuels of different levels of maturity [2]. Alkanes with different carbon numbers can distinguish natural sources from anthropogenic sources [5–7]. What is more, diagnostic ratios of tracers were also used to identify the sources [8]. Usually, V and Ni are considered as tracers of heavy fuel oil combustion, and they are employed as typical tracers of ship emissions when V/Ni and V/Pb are higher than 0.7 and 0.27 [9–10], respectively. The IcdP/(IcdP + BghiP) and Flt/(Flt + Pyr) are used to distinguish different fossil fuels[8, 11–12]. BaP degrades faster than BeP, which may be strongly influenced by photodegradation and long-distance transport [11, 13–14]. The lowest C29αβ/C30αβ value for hopane can also be used to judge fossil fuels [15]. The homohopane index C34[S/(S+R)] can distinguish fuel maturity, which is defined as:

Citation: Xue, Q.; Tian, Y.; Liu, X.; Wang, X.; Huang, B.; Zhu, H.; Feng, Y. Potential Risks of PM2.5-Bound Polycyclic Aromatic Hydrocarbons and Heavy Metals from Inland and Marine Directions for a Marine Background Site in North China. *Toxics* **2022**, *10*, 32. https://doi.org/10.3390/toxics10010032

Academic Editors: Matthias Karl; Yuan Cheng

Received: 22 November 2021
Accepted: 05 January 2022
Published: 11 January 2022

Publisher's Note: MDPI stays neutral with regard to jurisdictional claims in published maps and institutional affiliations.

Copyright: © 2022 by the authors. Submitted for possible open access publication under the terms and conditions of the Creative Commons Attribution (CC BY) license (https://creativecommons.org/licenses/by/4.0/).

$$C34[S/(S+R)] = \frac{\mathrm{C34\alpha\beta S}}{\mathrm{C34\alpha\beta S + C34\alpha\beta R}} \quad (1)$$

For n-alkanes, the carbon preference index (CPI) can reflect the comparison between natural and anthropogenic contributions, which is defined as the ratio of the total concentration of odd n-alkanes to that of even n-alkanes [16]:

$$\mathrm{CPI} = \frac{\sum_{i=5}^{16} C_{2i+1}}{\sum_{i=5}^{16} C_{2i}} \quad (2)$$

where i is the carbon number. Due to the fact that plant wax n-alkanes show strong odd carbon number predominance, biogenic n-alkanes should have CPI values greater than unity, whereas anthropogenic n-alkanes should have CPI values close to unity [16].

Lower values of C29/C17 for n-alkanes and terrigenous-to-aquatic ratios (TAR) implied increased contributions from aquatic inputs [16].

$$\mathrm{TAR=C27+C29+C31/C15+C17+C19} \quad (3)$$

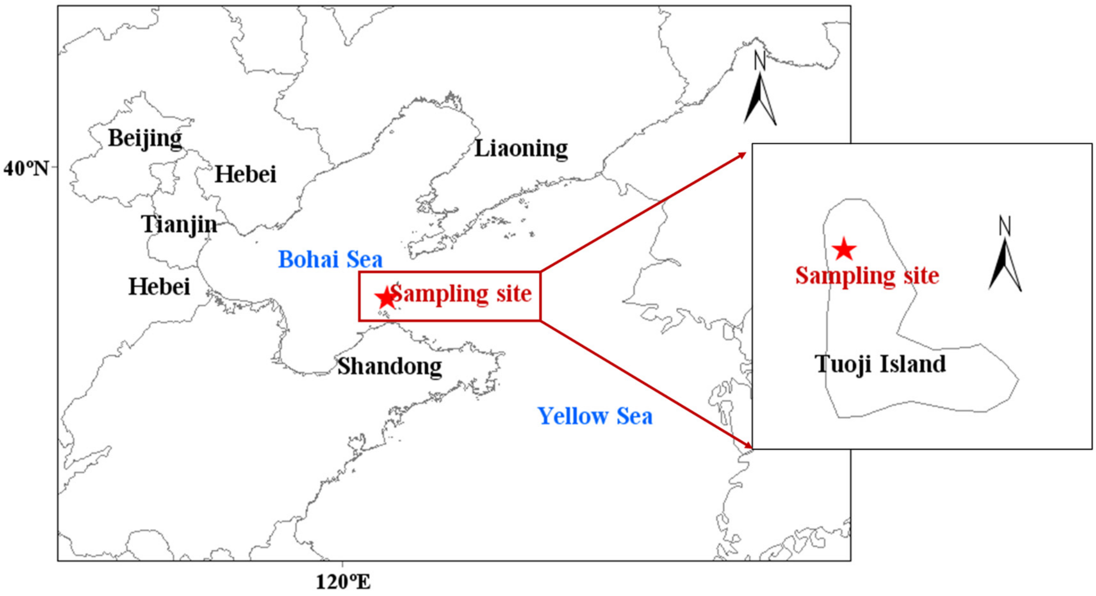

Figure S1. Location of the sampling site and the adjacent provinces and municipalities.

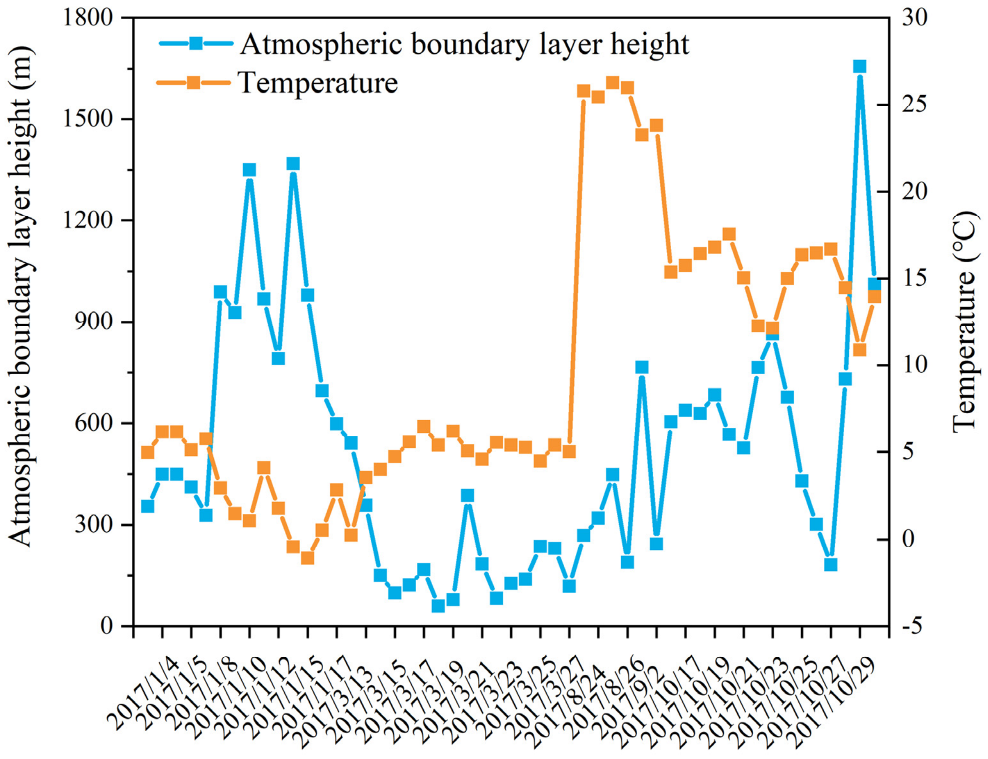

Figure S2. The atmospheric boundary layer height and temperature of the sampling site during the sampling periods.

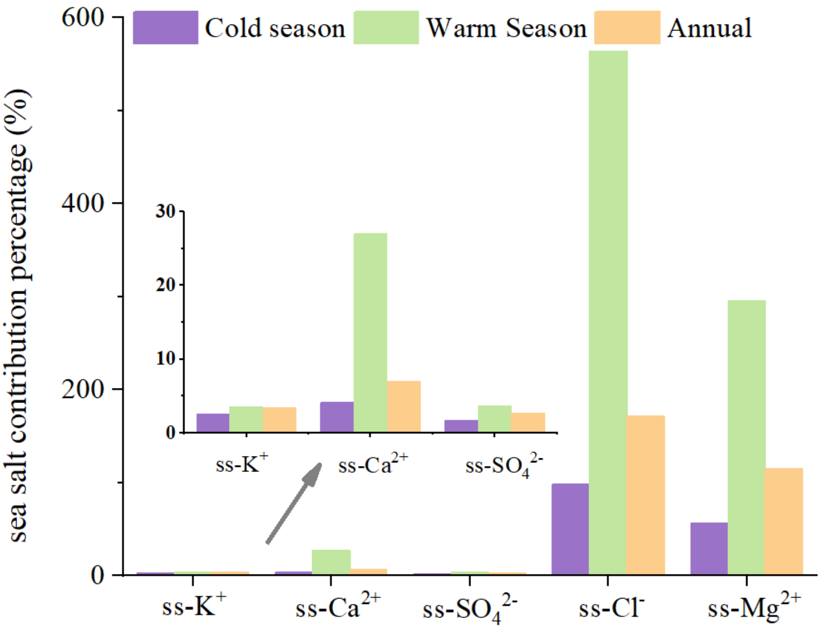

Figure S3. The warm and cold seasonal percentages of K^+, Mg^{2+}, Ca^{2+}, Cl^-, and SO_4^{2-} from sea salt accounting for total amount of the corresponding chemical species.

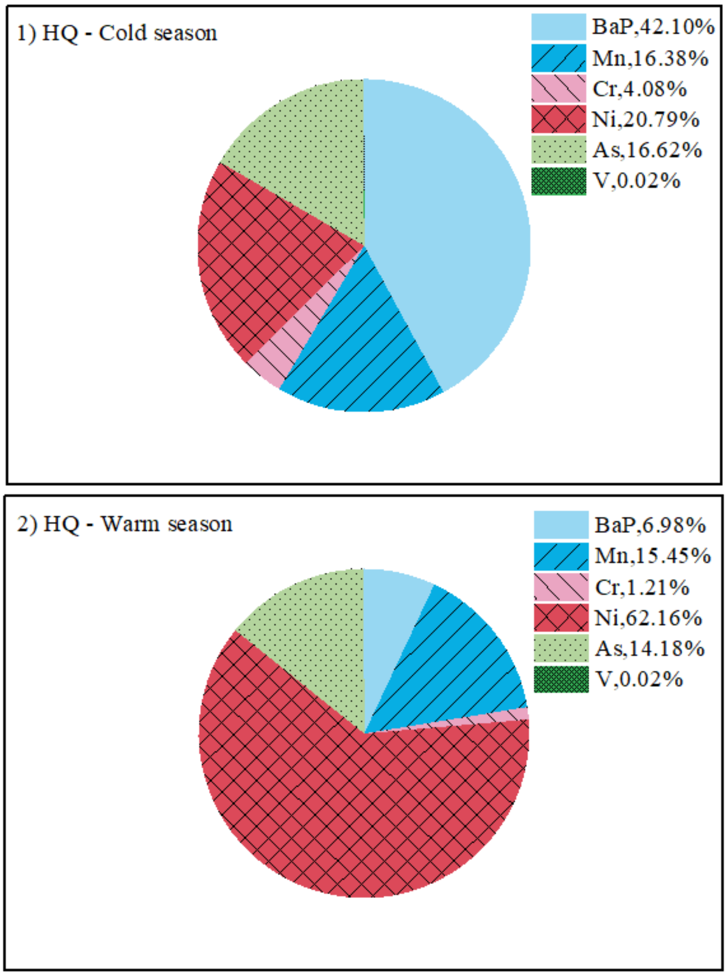

Figure S4. The warm and cold seasonal proportion of the HQ of each component (PAHs and HMs) relative to the total HQ.

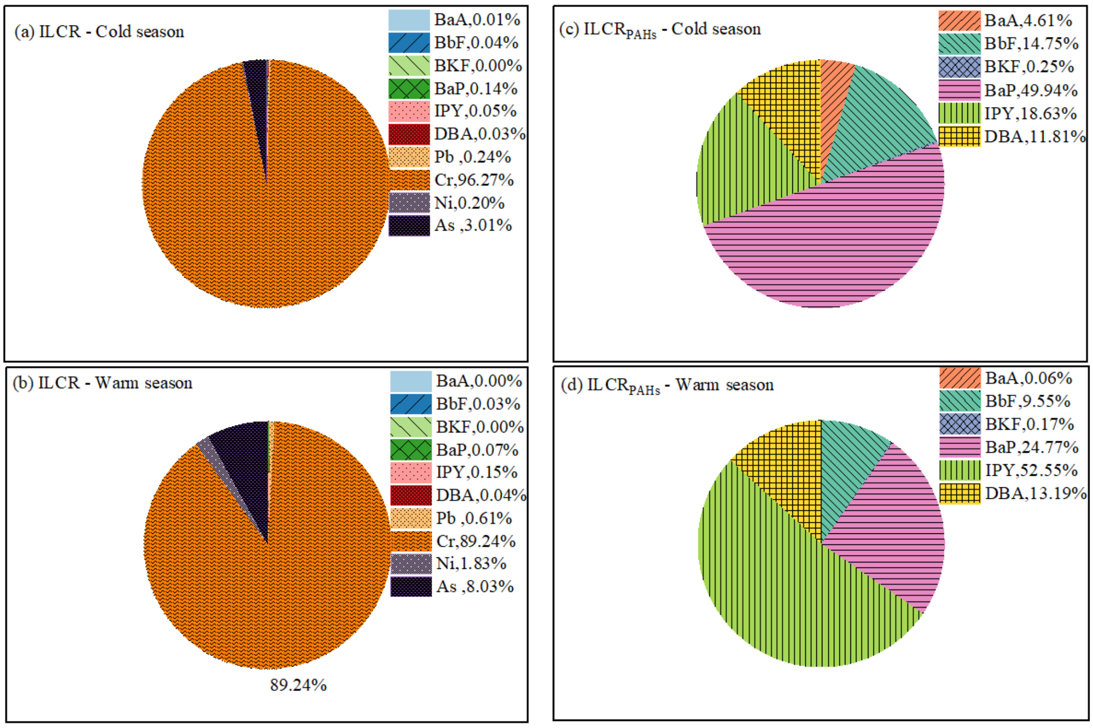

Figure S5. The warm and cold seasonal proportions of ILCR of each component (PAHs and HMs) relative to the total ILCR.

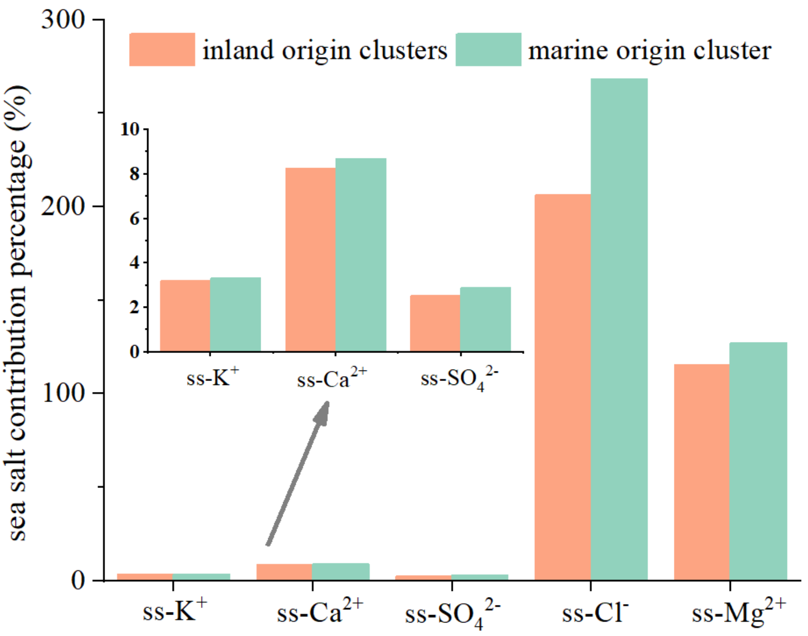

Figure S6. The percentages of K^+, Mg^{2+}, Ca^{2+}, Cl^-, and SO_4^{2-} from sea salt accounting for the total amount of the corresponding chemical species from inland- and marine-origin clusters.

Table S1. The detailed researched compounds for conventional and organic components.

Full name	Abbreviation	Manufacturer	Full name	Abbreviation	Manufacturer
Aluminum	Al	As-One, Japan	Indeno(1,2,3-cd)pyrene	IcdP	02si, USA
Arsenic	As	As-One, Japan	Dibenzo(a,h)anthracene	DBA	02si, USA
Calcium	Ca	As-One, Japan	Benzo(g,hi)perylene	BghiP	02si, USA
Cadmium	Cd	As-One, Japan	Coronene	Cor	02si, USA
Cobalt	Co	As-One, Japan	17α(H),21β(H)-(22R)-Tetrakishomohopane	C34αβS	02si, USA
Chromium	Cr	As-One, Japan	17α(H),21β(H)-(22S)-Tetrakishomohopane	C34αβR	02si, USA
Copper	Cu	As-One, Japan	17α(H)-22,29,30-Trisnorhopane™	C27α	02si, USA
Iron	Fe	As-One, Japan	17α(H),21β(H)-Hopane	C30αβ	02si, USA
Potassium	K	As-One, Japan	17β(H),21α(H)-Hopane	C30βα	02si, USA
Magnesium	Mg	As-One, Japan	17ß(H),21ß(H)-Hopane	C30ββ	02si, USA
Manganese	Mn	As-One, Japan	17α(H),21β(H)-30-Norhopane	C29αβ	02si, USA
Sodium	Na	As-One, Japan	ααα (20R)-Cholestane	C27αααR	02si, USA
Nickel	Ni	As-One, Japan	ααα (20S)-Cholestane	C27αααS	02si, USA
Lead	Pb	As-One, Japan	n-Tridecane	C13	02si, USA
Silicon	Si	As-One, Japan	n-Tetradecane	C14	02si, USA
Titanium	Ti	As-One, Japan	n-Pentadecane	C15	02si, USA
Vanadium	V	As-One, Japan	n-Hexadecane	C16	02si, USA
Zinc	Zn	As-One, Japan	n-Heptadecane	C17	02si, USA
Organic carbon	OC	As-One, Japan	n-Octadecane	C18	02si, USA
Elemental carbon	EC	As-One, Japan	n-Nonadecane	C19	02si, USA
Nitrate ion	NO_3^-	SIMS,ChIna	n-Eicosane	C20	02si, USA
Sulfate ions	SO_4^{2-}	SIMS,ChIna	n-Heneicosane	C21	02si, USA
Ammonium ion	NH_4^+	SIMS,ChIna	n-Docosane	C22	02si, USA
Chloride ion	Cl^-	SIMS,ChIna	n-Tricosane	C23	02si, USA
Sodium ion	Na^+	SIMS,ChIna	n-Tetracosane	C24	02si, USA
Magnesium ions	Mg^{2+}	SIMS,ChIna	n-Pentacosane	C25	02si, USA
Calcium ions	Ca^{2+}	SIMS,ChIna	n-Hexacosane	C26	02si, USA
Potassium ion	K^+	SIMS,ChIna	n-Heptacosane	C27	02si, USA
Naphthalene	Nap	02si, USA	n-Octacosane	C28	02si, USA
Acenaphthylene	Any	02si, USA	n-Nonacosane	C29	02si, USA
Acenaphthene	Ana	02si, USA	n-Tricontane	C30	02si, USA
Fluorene	Flu	02si, USA	n-Hentriacontane	C31	02si, USA
Phenanthrene	Phe	02si, USA	n-Dotriacontane	C32	02si, USA
Anthracene	Ant	02si, USA	n-Tritriacontane	C33	02si, USA
Fluoranthene	Flt	02si, USA	n-Tetratriacontane	C34	02si, USA
Pyrene	Pyr	02si, USA	n-Pentadecane	C35	02si, USA
Chrysene	Chr	02si, USA	n-Hexadecane	C36	02si, USA
Benzo(a)anthracene	BaA	02si, USA	n-Heptadecane	C37	02si, USA
Benzo(b)fluoranthene	BbF	02si, USA	n-Octacosane	C38	02si, USA
Benzo(k)fluoranthene	BkF	02si, USA	n-Nonacosane	C39	02si, USA
Benzo(a)pyrene	BaP	02si, USA	n-Tetradecane	C40	02si, USA
Benzo(e)pyrene	BeP	02si, USA			

SIMS: Shandong Institute of Metallurgical Sciences.

Table S2. The limit of detection (LOD) and limit of quantitation (LOQ) for conventional and organic components.

Name	LOD ($\mu g\ m^{-3}$)	LOQ ($\mu g\ m^{-3}$)	Name	LOD ($\mu g\ m^{-3}$)	LOQ ($\mu g\ m^{-3}$)	Name	LOD ($\mu g\ m^{-3}$)	LOQ ($\mu g\ m^{-3}$)
Al	0.0476	0.1904	K^+	0.05	0.2	C13	0.00199	0.00796
As	0.0064	0.0256	Nap	0.0002	0.0008	C14	0.01556	0.06224
Ca	0.2605	1.042	Any	0.00657	0.02628	C15	0.00552	0.02208
Cd	0.0003	0.0012	Flu	0.0006	0.0024	C16	0.03227	0.12908
Co	0.0008	0.0032	Phe	0.0005	0.002	C17	0.02142	0.08568
Cr	0.0007	0.0028	Ant	0.0004	0.0016	C18	0.02864	0.11456
Cu	0.0073	0.0292	Flt	0.0004	0.0016	C19	0.01334	0.05336
Fe	0.0416	0.1664	Pyr	0.0004	0.0016	C20	0.01836	0.07344
K	0.0191	0.0764	Chr	0.0007	0.0028	C21	0.00692	0.02768
Mg	0.0126	0.0504	BaA	0.0005	0.002	C22	0.02002	0.08008
Mn	0.0004	0.0016	BbF	0.0009	0.0036	C23	0.0217	0.0868
Na	0.021	0.084	BkF	0.0007	0.0028	C24	0.01376	0.05504
Ni	0.00033	0.00132	BaP	0.0009	0.0036	C25	0.01596	0.06384
Pb	0.0044	0.0176	BeP	0.0003	0.0012	C26	0.01218	0.04872
Si	0.0304	0.1216	IcdP	0.0009	0.0036	C27	0.0116	0.0464
Ti	0.0002	0.0008	DBA	0.0007	0.0028	C28	0.02412	0.09648
V	0.0008	0.0032	BghiP	0.0006	0.0024	C29	0.01316	0.05264
Zn	0.006	0.024	Cor	0.0001	0.0004	C30	0.01893	0.07572
OC	0.82	3.28	C34αβS	0.0001	0.0004	C31	0.01479	0.05916
EC	0.2	0.8	C34αβR	0.00015	0.0006	C32	0.01265	0.0506
NO_3^-	0.12	0.48	C27α	0.00013	0.00052	C33	0.0088	0.0352
SO_4^{2-}	0.34	1.36	C30αβ	0.00094	0.00376	C35	0.00212	0.00848
NH_4^+	0.25	1	C30βα	0.00148	0.00592	C37	0.00069	0.00276
Cl^-	0.11	0.44	C30ββ	0.00016	0.00064	C38	0.00136	0.00544
Na^+	0.06	0.24	C29αβ	0.00002	0.00008	C39	0.00037	0.00148
Mg^{2+}	0.06	0.24	C27αααR	0.00003	0.00012	C40	0.00024	0.00096
Ca^{2+}	0.26	1.04	C27αααS	0.00011	0.00044			

Table S3. Diagnostic ratios of tracer species.

	Gasoline vehicles	Diesel vehicles	Coal emission	Ship emission	Photo-degradation	Aquatic macrophytes and plankton	References	Cluster 1	Cluster 2	Cluster 3
OC/EC	High	Low	Low	Low			[2,17]	3.43	3.45	2.96
V/Ni				> 0.7			[9,10]	0.42	0.17	0.43
V/Pb				> 0.27			[9,10]	0.16	0.14	0.25
Cu/Zn	Low	High		High			[1]	0.15	0.13	0.15
Flt/(Flt+Pyr)	0.4–0.5	0.60–0.70	> 0.5				[11,12]	0.55	0.56	0.61
BaP/BeP					< 0.4		[13,14]	0.51	0.44	0.57
IcdP/(IcdP+Bghip)	0.12–0.22 (0.18)	> 0.3 (0.37)	(> 0.5) 0.58				[11,12]	0.26	0.41	0.29
Cmax	C23;C25	C17;C20		C17			[16]	C17	C18	C16
TAR	High	High	High	High	High	Low	[16]	0.33	0.41	0.43
C29/C17	High	High	High	High	High	Low	[16]	0.18	0.15	0.24
C34αβS/C34αβS + C34αβR	0.6	0.5	0.1–0.4				[15]	0.54	0.59	0.41
C29αβ/C30αβ	0.6–0.7	0.4	0.6–2.0				[15]	0.20	0.16	0.24
CPI			< 2				[16]	1.25	1.71	1.56

The terrigenous-to-aquatic ratio (TAR) was calculated by C27 + C29 + C31/C15 + C17 + C19.

Table S4. The parameters (EF, ED, BW, AT) used in CDI formulas.

Abbreviation	Full name	Unit	Adult	References
C	Concentration of components	$\mu g\ m^{-3}$		

InhR	Inhalation rate	m^3 day^{-1}	20	[17,18]
EF	Exposure frequency	Days $years^{-1}$		
ED	Exposure duration	Years	30	[19,20]
BW	Body weight	kg	70	[19,20]
$AT_{non\text{-}carcer}$	Average time	Days	10950	[19,20]
AT_{carcer}			25550	[19,20]

Table S5. The *SFi* values of the HMs and PAHs.

Name	RfDinh	SF	References
BaA	/	0.21	[19,20]
CHR	/	0	[19,20]
BbF	/	0.21	[19,20]
BkF	/	0.02	[19,20]
BaP	5.71×10^{-7}	2.1	[19,20]
DBA	/	2.1	[19,20]
IPY	/	0.21	[19,20]
Fe	/	42	[19,20]
Mn	1.43×10^{-5}	9.8	[19,20]
Cu	/	0.84	[19,20]
Zn	/	15.1	[19,20]
Pb	/	0.28	[19,20]
Cr	2.86×10^{-5}	294	[19,20]
Ni	4.00×10^{-6}	0.84	[19,20]
As	4.29×10^{-6}	15.05	[19,20]
V	2.86×10^{-3}	/	[19,20]

References:

1. Iakovides, M.; Iakovides, G.; Stephanou, E.G. Atmospheric particle-bound polycyclic aromatic hydrocarbons, n-alkanes, hopanes, steranes and trace metals: PM2.5 source identification, individual and cumulative multi-pathway lifetime cancer risk assessment in the urban environment. *Sci. Total Environ.* **2021**, *752*. 141834.
2. Esmaeilirad, S.; Lai, A.; Abbaszade, G.; Schnelle-Kreis, J.; Zimmermann, R.; Uzu, G.; Daellenbach, K.; Canonaco, F.; Hassankhany, H.; Arhami, M.; Baltensperger, U.; Prévôt, A.S.H.; Schauer, J.J.; Jaffrezo, J.-L.; Hosseini, V.; El Haddad, I. Source apportionment of fine particulate matter in a Middle Eastern Metropolis, Tehran-Iran, using PMF with organic and inorganic markers. *Sci. Total Environ.* **2020**, *705*, 135330.
3. Galvao, E.S.; Reis, Jr N.C.; Lima, A.T.; Stuetz, R.M.; D'Azeredo, O.M.T.; Santos, J.M. Use of inorganic and organic markers associated with their directionality for the apportionment of highly correlated sources of particulate matter. *Sci. Total Environ.* **2019**, *651*, 1332-1343.
4. Kang, M.J.; Ren, L.J.; Ren, H.; Zhao, Y.; Kawamura, K.; Zhang, H.L.; Wei, L.F.; Sun, Y.L.; Wang, Z.F.; Fu, P.Q. Primary biogenic and anthropogenic sources of organic aerosols in Beijing, China: Insights from saccharides and n-alkanes. *Environ. Pollut.* **2018**, *243*, 1579-1587.
5. Pereira, G.M.; Teinila, K.; Custodio, D.; Santos, A.G.; Xian, H.; Hillamo, R.; Alves, C.A.; de Andrade, J.B.; da Rocha, G.O.; Kumar, P.; Balasubramanian, R.; Andrade, M.D.F.; Vasconcellos, P.D.C. Particulate pollutants in the Brazilian city of Sao Paulo: 1-year investigation for the chemical composition and source apportionment. *Atmos. Chem. Phys.* **2017**, *17*, 11943-11969.
6. Souza, M.R.R.; Suzarte, J.S.; Carmo, L.O.; Santos, E.; Soares, L.S.; Júnior, A.R.V.; Santos, L.G.G.V.; Krause, L.C.; Damasceno, F.C.; Frena, M.; Alexandre, M.R. Assessment of polycyclic aromatic hydrocarbons in three environmental components from a tropical estuary in Northeast Brazil. *Mar. Pollut. Bull.* **2021**, *171*, 112726.
7. Vaezzadeh, V.; Yi, X.; Rais, F.R.; Bong, C.W.; Thomes, M.W.; Lee, C.W.; Zakaria, M.P.; Wang, A.J.; Zhong, G.C.; Zhang, G. Distribution of black carbon and PAHs in sediments of Peninsular Malaysia. *Mar. Pollut. Bull.* **2021**, *172*, 112871.
8. Tobiszewski, M.; Namieśnik, J. PAH diagnostic ratios for the identification of pollution emission sources. *Environ. Pollut.* **2012**, *162*, 110-119.
9. Zhang, F.; Chen, Y.J.; Tian, C.G.; Wang, X.P.; Huang, G.P.; Fang, Y.; Zong, Z. Identification and quantification of shipping emissions in Bohai Rim, China. *Sci. Total Environ.* **2014**, *497-498*, 570-577.
10. Mamoudou, I.; Zhang, F.; Chen, Q.; Wang, P.; Chen, Y. Characteristics of PM2.5 from ship emissions and their impacts on the ambient air: A case study in Yangshan Harbor, Shanghai. *Sci. Total Environ.* **2018**, *640-641*, 207-216.
11. Zhang, J.M.; Yang, L.X.; Mellouki, A.; Chen, J.M.; Chen, X.F.; Gao, Y.; Jiang, P.; Li, Y.Y.; Yu, H.; Wang, W.X. Diurnal concentrations, sources, and cancer risk assessments of PM2.5-bound PAHs, NPAHs, and OPAHs in urban, marine and mountain environments. *Chemosphere,* **2018**, *209*, 147-155.

12. Chen, R.; Jia, B.; Tian, Y.Z.; Feng, Y.C. Source-specific health risk assessment of PM2.5-bound heavy metals based on high time-resolved measurement in a Chinese megacity: insights into seasonal and diurnal variations. *Ecotox. Environ. Saf.* **2021**, *216*, 112167.
13. Jin, L.; Xie, J.W.; Wong, C.K.C.; Chan, S.K.Y.; Abbaszade, G.; Schnelle-Kreis, J.; Zimmermann, R.; Li, J.; Zhang, G.; Fu, P.Q.; Li, X.D. Contributions of City-Specific Fine Particulate Matter (PM2.5) to Differential In Vitro Oxidative Stress and Toxicity Implications between Beijing and Guangzhou of China. *Environ. Sci. Technol.* **2019**, *53*, 2881-2891.
14. Kong, S.F.; Yan, Q.; Zheng, H.; Liu, H.B.; Wang, W.; Zheng, S.R.; Yang, G.W.; Zheng, M.M.; Wu, J.; Qi, S.H.; Shen, G.F.; Tang, L.L.; Yin, Y.; Zhao, T.L.; Yu, H.; Liu, D.T.; Zhao, D.L.; Zhang, T.; Ruan, J.J.; Huang, M.Z. Substantial reductions in ambient PAHs pollution and lives saved as a co-benefit of effective long-term PM2.5 pollution controls. *Environ. Int.* **2018**, *114*, 266-279.
15. Tian, Y.Z.; Liu, X.; Huo, R.Q.; Shi, Z.B.; Sun, Y.M.; Feng, Y.C.; Harrison R.M. Organic compound source profiles of PM2.5 from traffic emissions, coal combustion, industrial processes and dust. *Chemosphere*, **2021**, *278*, 130429.
16. Lyu, Y.; Xu, T.T.; Yang, X.; Chen, J.M.; Cheng, T.T.; Li, X. Seasonal contributions to size-resolved n-alkanes C8–C40 in the Shanghai atmosphere from regional anthropogenic activities and terrestrial plant waxes. *Sci. Total Environ.* **2017**, *579*, 1918-1928.
17. Sun, Y.M.; Tian, Y.Z.; Xue, Q.Q.; Jia, B.; Wei, Y.; Song, D.L.; Huang, F.X.; Feng, Y.C. Source-specific risks of synchronous heavy metals and PAHs in inhalable particles at different pollution levels: Variations and health risks during heavy pollution. *Environ. Int.* **2021**, *146*, 106162.
18. US EPA. *Exposure Factors Handbook 2011* Edition (Final Report). U.S. Environmental Protection Agency, Washington, DC, EPA/600/R-09/052F, **2011**.
19. US EPA. *User's guide/technical background document for US EPA Region 9's RSL tables. Washington, DC,* US Environmental Protection Agency, **2013**.
20. Tian, Y.Z.; Li, Y.X.; Liang, Y.L.; Xue, Q.Q.; Feng, X.; Feng, Y.C. Size distributions of source-specific risks of atmospheric heavy metals: An advanced method to quantify source contributions to size-segregated respiratory exposure. *J. Hazard. Mater.* **2021**, *407*, 124355.
